# Genetic analysis of methicillin‐susceptible *Staphylococcus aureus* clinical isolates: High prevalence of multidrug‐resistant ST239 with strong biofilm‐production ability

**DOI:** 10.1002/jcla.23494

**Published:** 2020-07-21

**Authors:** Hossein Goudarzi, Mehdi Goudarzi, Fattaneh Sabzehali, Maryam Fazeli, Alireza Salimi Chirani

**Affiliations:** ^1^ Department of Microbiology School of Medicine Shahid Beheshti University of Medical Sciences Tehran Iran; ^2^ Department of Virology Pasteur Institute of Iran Tehran Iran

**Keywords:** *agr* allotype, Biofilm, Methicillin‐susceptible *S aureus*, multilocus sequence typing, *Staphylococcus aureus*

## Abstract

**Background:**

The distributions of methicillin‐susceptible *Staphylococcus aureus* (MSSA) are divers geographically with different genetic backgrounds. Data related to molecular characteristics of MSSA compare to methicillin‐resistant *Staphylococcus aureus* (MRSA) is sparse.

**Methods:**

In this cross‐sectional study, antimicrobial susceptibility testing, virulence genes analysis, biofilm formation, accessory gene regulator (*agr*) typing, and multilocus sequence typing (MLST) characterized on 75 MSSA isolates.

**Results:**

Multidrug‐resistance MSSA was found to be 84%. Forty‐eight (64%) isolates were toxinogenic with 34 and 14 isolates carrying *pvl* and *tst* representing 45.3% and 18.7%. The most common SE genes were *sed* (20%), *sec* (16%), and *sea* (16%). Fifty‐five (73.3%) isolates were confirmed as biofilm producer with a markedly high prevalence of *fnbA* (93.3%), *fnbB* (86.7%), *icaA* (65.3%), *icaD* (53.3%), *can* (24%), *ebp* (10.7%), and *bap* (1.3%). A total of 3 *agr* types (I, 73.3%; III, 16%; II, 10.7%) and 4 clonal complexes (CCs) and sequence types (STs), namely CC8/ST293 (45.3%), CC/ST22 (28%), CC/ST30 (16%), and CC/ST5 (10.7%) were detected in this study. All the high and low‐level mupirocin resistance strains belonged to ST239 and ST22 strains, respectively. All the fusidic acid‐resistant isolates carried *fusC* and belonged to ST30.

**Conclusions:**

These findings indicated that ST239 with strong biofilm production ability is the most common type in MSSA strains isolated from patients. It seems that the antimicrobial resistance profiles, toxin, and biofilm formation were closely associated with specific STs. Further studies are required to identify and control of these clonal lineages in our area.

## Graphical abstract

Biofilm formation involves first, adhesion to surfaces mediated by the expression of different microbial surface components recognizing adhesive matrix molecules (MSCRAMMs) and second, production of polysaccharide intercellular adhesion (PIA) which mediates cell to cell adhesion. Biofilm protects the bacteria from host defenses and prevents delivery of antibiotics and have an adverse effect on the eradication of infection. Several researchers noted concerns about biofilm formability on biotic and abiotic surfaces especially medical devices, by MSSA strains. It seems that biofilm formability in MSSA strains is most frequently associated with specific clonal lineages. There is a need for the continuous monitoring of these genotypes.

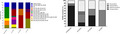

## 1 INTRODUCTION

*Staphylococcus aureus* as a major human pathogen in both hospitals and community settings is responsible for a variety of diseases ranging from skin and soft‐tissue infections (SSTIs), gastrointestinal disorders, and pneumonia to serious and life‐threatening diseases.^1^, ^2^, ^3^ Some of researchers from Asia reported a dramatic increase in the annual rate of visits for staphylococcal infections to healthcare settings which consequently represents a special concern related to the rate of mortality and morbidity.^4^, ^5^ However, it is well established that several infections are caused by methicillin‐susceptible *S aureus* (MSSA).^6^, ^7^ According to the evidence, variable rates of staphylococcal infections caused by MSSA have been reported in previously published data from Iran, ranged from 23.1% to 76.4%.^8^ Nowadays, infection associated with MSSA is a major public health crisis representing a priority for healthcare settings.^2^, ^3^, ^8^ Compelling evidence has indicated that virulence genes may play an important role in serious infections related to MSSA, and it is further exacerbated by widening resistance to currently available antibacterial agents.^9^, ^10^ Emerging simultaneous resistance to multiple antibacterial agents among MSSA strains has significantly limited the availability of chemotherapeutic agents for the treatment of staphylococcal infections and leads to deterioration of the disease.^11^, ^12^, ^13^, ^14^ *S aureus* produces virulence determinants including adhesions (collagen‐binding protein, clumping factor, fibronectin‐binding protein, and elastin‐binding protein), toxins (toxic shock syndrome toxin‐1, panton‐valentine leukocidin, exfoliative), enterotoxins (SEs), staphylokinase hemolysin, and lipase which are related to the severity of the infection.^15^, ^16^, ^17^ Several investigators noted concerns about biofilm formability on biotic and abiotic surfaces especially medical devices, by MSSA strains.^8^, ^18^, ^19^ Furthermore, biofilm formability can play a key role in the development of resistance, unsuccessful eradication of infection, and resistance to host immune response.^8^ MSSA isolates usually present fast dissemination and high genetic variability which makes their epidemiological understanding more complex.^2^, ^3^, ^4^, ^6^, ^10^, ^15^ Based on the evidence, MSSA isolates could be a reservoir for MRSA clones and are essential for controlling the potential emergence of new epidemic MRSA clones.^6^, ^20^ Therefore, gaining adequate knowledge about genotypic characteristics, understanding of the resistance and virulence pattern and the ability of biofilm formation is helpful to prevent and control the spread of MSSA strains. According to the published data characteristics of MSSA strains in Iran remain poorly understood. the current study was performed to investigate the resistance pattern, biofilm‐forming ability, and the presence of virulence factors, biofilm, and adhesion genes. Multilocus sequence typing (MLST), accessory gene regulator (*agr*) typing were used to characterize the genotype of the MSSA strains.

## 2 MATERIALS AND METHODS

### 2.1 Study design, identification of isolate and ethics statement

From January and August 2019, 75 MSSA isolates obtained from hospitalized patients in three hospitals affiliated to Shahid Beheshti University of Medical Sciences, Tehran, Iran were collected in the study. Sources of isolation included wound (25; 33.4%), abscess (16; 21.3%), respiratory tract secretions (11; 14.7%), urine (10; 13.3%), blood (6; 8%), conjunctivitis (4; 5.3%), and body fluids (3; 4%). Healthcare–associated MSSA (HA‐MSSA) infection was defined if the positive culture of MSSA was obtained on or after the third day of admission to a hospital.^21^ The Ethics Committee of the Shahid Beheshti University of Medical Sciences in Tehran, Iran approved this research (IR. SBMU. MSP.REC. 1398. 818). All isolates were confirmed as *S aureus* by using standard microbiological methods as well as polymerase chain reaction (PCR) assay for the presence of the *nuc* gene as described previously.^22^ The *S aureus* isolates susceptible to cefoxitin disc (30 µg, Mast Co., UK) and negative for the presence of *mecA* gene by PCR were considered as MSSA strains.^20^, ^22^

### 2.2 Evaluation of antimicrobial activities

Susceptibility to 16 antimicrobials including penicillin (PEN), amikacin (AMK), gentamicin (GEN), tobramycin (TOB), kanamycin (KAN), tetracycline (TET), erythromycin (ERY), clindamycin (CLI), linezolid (LIN), teicoplanin (TEC), ciprofloxacin (CIP), rifampicin (RIF), quinupristin‐dalfopristin (SYN), and trimethoprim‐sulfamethoxazole (SXT) (Mast Co., UK) was determined by Kirby–Bauer disk diffusion method in accordance with clinical laboratory standards institute (CLSI) guidelines (CLSI 2019).

The broth dilution method was applied to estimate minimal inhibitory concentrations (MIC) for vancomycin (VAN), mupirocin (MUP), tigecycline (TIG), and fusidic acid (FUS). Susceptibility testing for FUS and TIG was carried out according to the European Committee for antimicrobial susceptibility testing (EUCAST) recommendations (http://www.eucast.org). The inducible macrolide‐lincosamide‐streptogramin group B (iMLS_B_) resistance was determined by D‐test (CLSI 2019). Isolates showing resistance to erythromycin while being susceptible to clindamycin with no blunting zone were classified as the MS resistance phenotype. Resistance to both erythromycin and clindamycin was considered as constitutive (cMLS_B_) resistance phenotype. Low‐level and high‐level mupirocin resistance (LLMUPR, HLMUPR) *S aureus* isolates were identified based on the CLSI guideline (CLSI 2019). Test performance was monitored using *S aureus* ATCC 25923, ATCC 43300, and ATCC 29213 strains.

### 2.3 Phenotypic analysis of biofilm formation

Biofilm production was assessed by microtiter plate assay as previously described by Yousefi et al^23^ In this method biofilm formation was classified in 4 groups based on the optical density (OD) measured by ELISA reader at a wavelength of 490 nm as below: (a) strong, (b) moderate, (c) weak or (d) biofilm non‐producer strains. For quality control, *Staphylococcus epidermidis* ATCC 35984 strain as a biofilm producer was used in each run.

### 2.4 DNA isolation and resistance, virulence and biofilm genes analysis by PCR‐based assays

Genomic DNA was isolated using the phenol‐chloroform extraction method with a slight modification including adding 5 μL of 5 mg/mL of lysostaphin (Sigma‐Aldrich) to cell suspension and incubation at 37°C for 30 minutes to 1 hour.^24^ The quantity of DNA was adjusted approximately to 100 ng/μL which evaluated by a NanoDrop‐2000 spectrophotometer (Thermo Fisher Scientific). All of the isolates were screened for resistance encoding genes namely: *mecA, mecC, vanA, vanB, mupB, mupA, fusA, fusB, fusC, msr(A), msr(B), erm*(A), *erm*(B), *erm*(C), *tetM, tetL*, *tetO*, *tetK*, *ant* (4′)*‐Ia, aac* (6′)*‐Ie/aph* (2″), and *aph* (3′)*‐IIIa* and virulence encoding genes including SEs genes namely: *sea*, *seb*, *sec*, *sed*, *see*, *seg*, *seh*, *sei*, and *sej*; exfoliative toxin genes (*eta* and *etb*), Panton‐Valentine leukotoxin gene (*pvl*), and toxic shock syndrome toxin (*tst*) genes by PCR assay with oligonucleotide primers as previously described.^24^, ^25^, ^26^, ^27^ We also used PCR assay to assess biofilm by the presence of *icaA*, *icaB*, *icaC*, *icaD*, *can, ebp, fnbB, fnbA,* and *bap* genes.^19^, ^23^

### 2.5 Molecular typing methods

#### 2.5.1 *agr* typing

*Staphylococcus aureus* isolates underwent *agr* typing to determine specificity groups. Multiplex PCR‐based protocol was used for amplification of the hypervariable domain of *agr* locus. *agr* types I, II, III, and IV were expected to produce 441‐bp, 575‐bp, 323‐bp, and 659‐bp fragments, respectively.^28^

#### 2.5.2 MLST

*Staphylococcus aureus* isolates were further characterized by MLST and by amplifying and sequencing seven housekeeping genes (*pta*, *arcC*, *tpi*, *aroE*, *gmk*, *yqiL*, and *glpF)*. Sequence types (STs) were determined by the submission of the allelic profile to the online MLST database website (https://pubmlst.org/).

## 3 RESULTS

**Figure 1:**
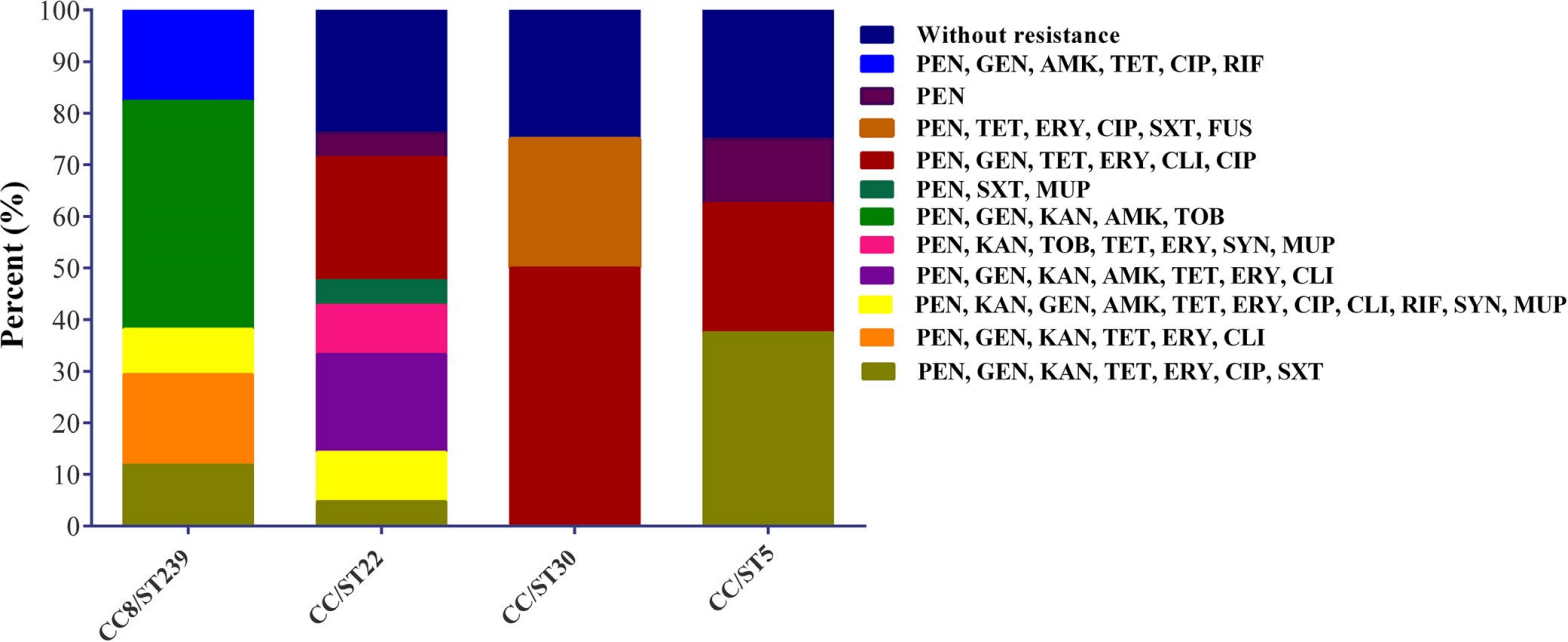
Distribution of different resistance profiles among healthcare‐associated methicillin‐susceptible *Staphylococcus aureus* (MSSA) clones

A total of 75 MSSA isolates belonged to 52 males and 23 females representing 69.3% and 30.7% of isolates were enrolled in this study. Out of 75 MSSA isolates, 31 isolates were obtained from hospital H1 (41.3%), 25 isolates from hospital H2 (33.3%), and 19 isolates from hospital H3 (25.3%). According to disc diffusion, the most frequently resistance was observed to penicillin (86.7%), followed by gentamicin (76%), tetracycline (68%), erythromycin (54.7%), kanamycin (53.3%), amikacin (48%), ciprofloxacin (46.7%), clindamycin (37.3%), tobramycin (22.7%), trimethoprim‐sulfamethoxazole (16%), rifampicin (14.7%), mupirocin (10.7%), quinupristin‐dalfopristin (9.3%), and fusidic acid (4%). Totally, eleven resistance patterns were identified, wherein PEN, GEN, KAN, AMK, TOB (20%, 15/75), PEN, GEN, TET, ERY, CLI, CIP (17.3%, 13/75) and PEN, GEN, KAN, AMK, TET, ERY, CLI (13.3%, 10/75) were the top three frequently identified patterns (Figure 1). None of the isolates were resistant to vancomycin, linezolid, teicoplanin. The MIC of vancomycin was 0.5 μg/mL for 15 isolates (20%), 1 μg/mL for 25 isolates (33.3%) and 2 μg/mL for 35 isolates (46.7%). Out of 8 mupirocin‐resistant MSSA isolates, 3 (4%) and 5 (6.7%) were HLMUPR and LLMUPR, respectively. All the HLMUPR isolates had MIC ≥512 µg/mL and harbored the *mupA* gene. Of 5 LLMUPR strains, 2 isolates (40%) were inhibited by 16 µg/mL of mupirocin, one isolate (20%) by 32 µg/mL, and two isolates (20%) by 64 µg/mL. None of the mupirocin‐resistant isolates harbored the *mupB* gene. The inducible cMLS_B_ and iMLS_B_ and MS phenotypes were detected in 22 (29.3%), 16 (21.3%), and 3 (4%) of the isolates, respectively. All the MSSA strains with iMLS_B_ phenotype harbored *erm*(C). Three isolates with MS phenotype did not harbor *erm* or *msr* genes. Out of 22 isolates with cMLS_B_ phenotype, *erm*(A) was detected in 20 isolates (71.4%) and *msr*(A) in 5 isolates (17.9%). Three isolates were fusidic acid‐resistant isolates with MICs of 8 μg/mL (2 strains) and 32 μg/mL (1 strain), carried all *fusC* and were isolated from wound (2 isolates) and abscess (1 isolate). The analysis of aminoglycoside resistance encoding genes indicated that the most prevalent gene was *ant* (4′)*‐Ia* (48%), followed by *aac* (6′)*‐Ie/aph* (2″) (26.7%), and *aph* (3′)*‐IIIa* (16%). Twenty‐five isolates (33.3%) contained *tetM*, 12 (16%) possessed *tetK*. Multidrug resistant (MDR) was defined if resistant to equal and more than three classes of antibiotics were observed among MSSA isolates^25^ which were 84% (63/75). Our findings displayed that no PCR products for the resistance genes *vanA, vanB, mupB, fusA, fusB, erm*(B), *mecC,* and *msr*(B) were observed.

Of 75 MSSA examined isolates, 48 (64%) were toxinogenic with 34 and 14 isolates carrying *pvl* and *tst* representing 45.3% and 18.7%. The most common SE genes were *sed*, *sec*, and *sea* which accounted for (20%; 15/75), (16%; 12/75), and (16%; 12/75), respectively. None of the isolates presented the *seb*, *see*, *eta*, *etb*, *seg*, *seh*, *sei,* and *sej* genes. Biofilm production analysis exhibited that 55 out of 75 MSSA isolates were biofilm producers (73.3%), from which 35 (63.6%) showed strong ability, 15 (27.3%) showed moderate ability, 5 (9.1%) showed weak ability in biofilm production. Genetic study of biofilm and adhesion genes revealed that *fnbA* as the most prevalent gene was present in 70 strains (93.3%), *fnbB* in 65 (86.7%), *icaA* in 49 (65.3%), *icaD* in 40 (53.3%) *can* in 18 (24%), *ebp* in 8 (10.7%), and *bap* in 1 (1.3%) isolates, respectively.

Overall, *agr* type I was identified as the predominant type, accounting for 73.3% (55/75) of all MSSA isolates, followed by type III (16%, 12/75), and type II (10.7%, 8/75). In present research, no isolates belonging to *agr* type IV was detected. MLST results showed that MSSA isolates were assigned to 4 clonal complexes (CCs) and sequence types (STs), namely CC8/ST293 (45.3%, 34/75), CC/ST22 (28%, 21/75), CC/ST30 (16%, 12/75), and CC/ST5 (10.7%, 8/75).

**Figure 2:**
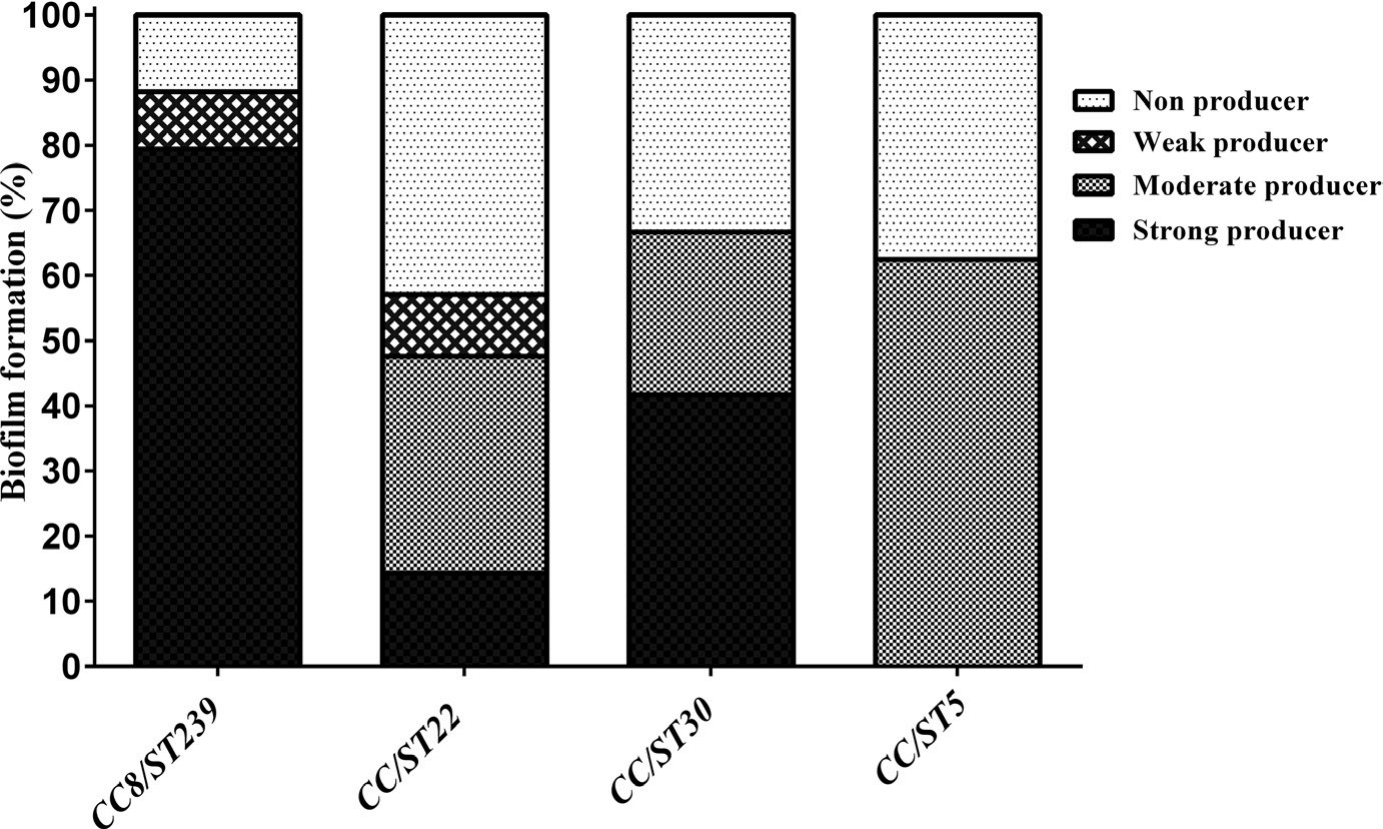
Biofilm formability of methicillin‐susceptible *Staphylococcus aureus* (MSSA) clones using the Microtiter Plate method based on the optical density

All HLMUPR strains belonged to ST239 (n = 3) while LLMUPR strains belonged to ST22 (n = 5) strains. Fusidic acid‐resistant isolates belonged to ST30 (n = 3). Isolates with iMLS_B_ phenotype were observed in CC88/ST239 (13.3% [10/75]), CC/ST22 (4% [3/75]), and CC/ST30 (4% [3/75]). Out of 22 MSSA strains with cMLS_B_ phenotype, 11 isolates belonged to CC/ST22 (14.7%), 6 isolates to CC/ST30 (8%), 3 isolates to CC8/ST239 (4%), and 2 isolates to CC/ST5 (2.7%). All 3 isolates with MS phenotype were belonged to ST5. Distribution of different resistance profiles among HA‐MSSA clones is presented in Figure 1. Our results revealed 55 (73.3%) strains were biofilm producers and 20 (26.7%) were non‐biofilm producers. Biofilm producer isolates were assigned to ST239 (54.6%, 30/55), ST22 (21.8%, 12/55), ST30 (14.5%, 8/55), and ST5 (9.1%, 5/55). Of the 75 HA‐MSSA isolates, 20 (26.7%) were confirmed as non‐biofilm producer isolates, which the majority of these isolates belonged to ST22 (45%), followed by ST239 (20%), ST30 (20%), and ST5 (15%). Figure 2 presented the distribution of CCs among biofilm producer and non‐biofilm producer strains.

The *bap* gene was detected in ST239 isolates. According to our analysis *icaA,* and *icaD* genes were present in all of the isolates with different percentages. The enterotoxin gene *sea* was detected in 4 ST239 isolates (5.3%), and 8 ST30 isolates (10.7%). Among the 12 second‐positive MSSA isolates, ST22 was the dominant clone (10.7%) and followed by ST30 (5.3%). The enterotoxin gene *sed* was observed in isolates with ST239 (13.3%) and ST5 (6.7%). We found a great number of ST22 MSSA strains (76.2%) that carried the *pvl* gene. Table 1 gives information about the characterization of MSSA strains obtained from clinical samples.

**Table 1:** Distribution of STs, biofilm ability, and molecular characterization of MSSA strains

CC	MLST	*agr* type	Biofilm status (No; %)	Antibiotic resistance genes (No; %)	Virulence/biofilm genes (No; %)	Hospitals (No; %)	Total N (%)
CC8	ST239	I	Producer (30; 88.2), Non‐producer (4; 11.8)	*mupA* (3; 8.8), *erm(A)* (9; 26.5), *erm(C)* (4; 11.8), *ant (4*′*)‐Ia* (16; 47.1), *aac (6*′*)‐Ie/aph (2*″*)* (8; 23.5), *aph (3*′*)‐IIIa* (4; 11.8), *tetM* (12; 35.3), *tetK* (7; 20.6)	*pvl* (8; 23.5), *tst* (6; 17.6), *sea* (4; 11.8), *sed* (10; 29.4)/ *icaA* (20; 58.8), *icaD* (15; 44.1), *fnbB* (34; 100), *fnbA* (34; 100), *bap* (1; 2.9), *ebp* (6; 17.6)	H1 (15; 44.1), H2 (10; 29.4), H3 (9; 26.5)	34 (45.3)
CC22	ST22	I	Producer (12; 57.1), Non‐producer (9; 42.9),	*msr(A)* (5; 23.8), *erm(C)* (3; 14.3), *ant (4*′*)‐Ia* (12; 57.1), *aac (6*′*)‐Ie/aph (2*″*)* (12; 57.1), *aph (3*′*)‐IIIa* (2; 9.5), *tetK* (5; 23.8)	*pvl* (16; 76.1), *tst* (3; 14.3), *sec* (8; 38.1)/ *icaA* (15; 71.4), *icaD* (15; 71.4), *fnbA* (16; 76.2), *fnbB* (15; 71.4), *can* (10; 47.6), *ebp* (2; 9.5)	H1 (10; 47.6), H2 (5; 23.8), H3 (6; 28.6)	21 (28)
CC30	ST30	III	Producer (8; 66.7), Non‐producer (4; 33.3)	*erm(A)* (11; 91.7), *fusC* (3; 25), *ant (4*′*)‐Ia* (6; 50), *aph (3*′*)‐IIIa* (3; 25), *tetM* (10; 83.3)	*pvl* (10; 83.3), *sea* (8; 66.7), *sec* (4; 33.3)/ *icaA* (8; 66.7), *icaD* (6; 50), *fnbB* (10; 83.3), *fnbA* (12; 100), *can* (5; 41.7)	H1 (5; 41.7), H2 (6; 50), H3 (1; 8.3)	12 (16)
CC5	ST5	II	Producer (5; 62.5), Non‐producer (3, 37.5)	*erm(C)* (3; 37.5), *ant (4*′*)‐Ia* (2; 25), *aph (3*′*)‐IIIa* (4; 50), *tetM* (3; 37.5)	*tst* (5; 62.5), *sed* (5; 62.5)/*icaA* (6; 75), *icaD* (4; 50), *fnbA* (8; 100), *fnbB* (6; 75), *can* (3; 37.5)	H1 (1; 12.5), H2 (4; 50), H3 (3; 37.5)	8 (10.7)

Abbreviations: MLST, multilocus sequence typing; MSSA, methicillin‐susceptible *Staphylococcus aureus*.

## 4 DISCUSSION

Infections caused by MSSA in both community and healthcare setting is increasing and has gained great attention in recent years.^2^, ^8^, ^16^, ^19^ The present research had several findings including diverse genetic backgrounds of MSSA isolates with a predominance of C8/ST239 associated with lineages commonly observed among MRSA suggesting that MRSA probably originated from MSSA clones. High biofilm formation ability in clonal lineages of MSSA was observed (73.3%). A relatively high *pvl* positive MSSA strains were noted that may reflect the important role of these isolates as reservoirs for *pvl* positive MRSA clones.

Data relating to susceptibility testing revealed that 10.7% of MSSA isolates were mupirocin resistant. This finding was differing from the previous study from other regions such as Iran (3%)^13^ and South Africa (3%).^14^ There are several studies that exhibited a high prevalence of mupirocin resistance among MRSA isolates comparing MSSA isolates. Conversely, our reported rate was higher than those reported previously among MRSA strains obtained from Iran.^13^ A recent systematic review and meta‐analysis conducted by Dadashi et al^29^showed that the pooled prevalence of mupirocin resistance and HLMUPR MRSA clinical isolates was 13.8% and 8.1%, respectively, in different parts of world that is higher than our reported rate. The reasons for resistance to mupirocin in present study could be injudicious and widespread use, uncontrolled policies in the prescription of this antibiotic, easy access to this antibiotic without prescription, inexpensive drugs, and spreading of specific lineage in these area.

Furthermore, we also observed that 3 (4%) and 5 (6.7%) isolates were HLMUPR and LLMUPR, respectively. In the study performed by Abdulgader et al^14^ in South Africa on 212 *S aureus* isolates, 1% and 3% of the studied MSSA isolates indicated high and low levels of resistance to mupirocin. The reasons for discrepancies in rates of resistance to mupirocin could be studied population, spreading of specific lineage, differences in infection control strategies and unrestricted policies in the prescription of this antibiotic. The current survey displayed that all HLMUPR‐MSSA strains carried *mupA* gene. In the study conducted in South Africa, a similar result was observed.^14^ In our study, neither *mupA* nor *mupB* was detected in the LLMUPR isolates which is consistent with previous studies.^13^, ^14^

In the current study, the prevalence of iMLSB was found to be 21.3%, which was higher than those reported in Nepal (4.95%),^30^ Brazil (5.8%),^11^ India (9.3%)^31^ and was near to reported rate in Ethiopia (21.4%).^32^ In line with several studies performed in other parts of the world, we found a higher prevalence of cMLSB phenotype compare to iMLSB phenotype (29.3% vs 21.3%).^11^, ^31^, ^32^ In the contrary, Bottega et al^11^ reported a low prevalence of cMLSB phenotype among MSSA isolates compare to iMLSB phenotype. (3.6% vs 5.8%). However, high rates of cMLSB among MSSA isolates were also noted in other studies conducted in Nepal (20.8%),^30^ and India (13.38%).^31^ These dissimilarities could explain the different patterns of consumption and prescription of macrolides, ketolides, and circulation of specific types in communities and healthcare settings.

The *erm*(C) gene was present in iMLSB MSSA strains. This finding was comparable to a study conducted by Aqel et al^33^ which demonstrated *erm(A)* and *erm(C)* genes in majority of isolates. According to the previous reports, *erm(A)* and *erm (C)* genes were the main resistant genes responsible for constitutive resistance *S aureus* isolates. cMLS_B_ isolates contained *erm(A)*, and *msr(A)* genes accounting for 71.4%, and 17.9%. From Iran, Khodabandeh et al^34^ reported, *erm(A)* gene as the most common gene in cMLS_B_ *S aureus* strains (25.9%). We did not find any isolates carry *erm(B)* gene. This was in agreement with the research performed in Iran by Moosavian et al^35^ In contrast, high prevalence of *erm*(B) gene noted by Khodabandeh et al^34^ (11.3%).

Recently published data highlighted variabilities in fusidic acid resistance in both MRSA and MSSA strains. It was notable that resistance to fusidic acid was detected in 4% of MSSA isolates which carried *fusC*. Different prevalence of fusidic acid resistance has been reported from Greece (62.4%), Ireland (19.9%), Australia (7%), Canada (7%).^36^, ^37^ In Iran, the rate of fusidic acid resistance, during a 4‐year period on 726 tested *S aureus* isolates, was found to be 3%.^38^ According to the evidence low level of resistance to fusidic acid is generally caused by the horizontally transferable genes including *fusB*, *fusC,* and *fusD*. On the contrary, a study conducted in China illustrated fusidic acid resistance related to *fusB* gene in 10.5% of examined isolates while *fusC* and *fusA* genes were not present in any of the tested isolates.^12^ This finding supports the presence of *fusC* as the main determinant responsible for resistance to fusidic acid among MSSA isolates in Iran.

Our data displayed the prevalence of aminoglycoside resistance genes including *ant* (4′)*‐Ia*, *aac* (6′)*‐Ie/aph* (2″), and *aph* (3′)*‐IIIa* in 48, 26.7, and 16% of isolates. A previous study in Iran on 105 *S aureus* isolates reported the most common aminoglycoside resistance genes were *ant (4*′*)‐Ia* (94.7%), *aac (6*′*)‐Ie/aph (2*″*)* (81.1%), and *aph (3*′*)‐IIIa* (31.6%).^25^ These dissimilarities could explain the different patterns of consumption and prescription of aminoglycosides and geographical differences in the occurrence of aminoglycoside resistance genes.

In our study, 34 (45.3%) and 14 (18.7%) isolates carried *pvl* and *tst*. Several other recent studies from Africa (57%),^15^ Russia (55%),^10^ China (34.4%),^6^ Ireland (17%),^36^ and Lebanon (12%)^4^ confirmed *pvl* positive MSSA strains in their studies. Indeed, our study proposed a high potential of MSSA strains to secrete toxins, specially *pvl* which may finally evolve into MRSA. A recent systematic review and meta‐analysis study in Iran notified a relatively high prevalence of *tst* encoding gene among *S aureus* clinical isolates (21.3%) with an average range from 0% to 68%.^39^ A high prevalence of *tst* gene among MSSA isolates comparing MRSA isolates was noted by Motamedifar et al.^40^

A high prevalence of SEs genes was observed in the present study (52%). it was similar to 62.6% prevalence SEs genes in a study of Turkey. The Turkish study also indicated that the *sec* and *sed* genes were the most abundant toxin genes in clinical *S aureus* isolates.^41^ Similarly, *sed* (20%), *sec* (16%), and *sea* (16%) were the predominant SE gene detected in the current study. Similar to the previous reports from different countries, we found *see*, *eta*, *etb*, *seh*, *sei,* and *sej* genes in none of the isolates.^42^, ^43^ A 2015 Chinese study, Yu et al^43^ indicated that SEs genes detected in MSSA strains were including *sea* (31%), *seo* (28.2%), *seg* (22.5%), *sen, sec,* and *sei* (21.1%), *sem* (19.7%), *seb*, *sed,* and *seh* (9.9%).

According to the evidence, virulence factors, biofilm formation, and subsequently antibiotic resistance regulated by *agr* system, which is strongly linked with specific clonal lineages. In present work, *agr* type I was identified as the predominant type, followed by type III and type II as previously reported in some studies.^3^, ^10^ Several investigators were highlighted the key role of biofilm and adhesion‐related genes in biofilm formation.^18^, ^44^ In our strain collection, 84.7% of strains were biofilm producers. This finding was contrary to previously published data, that exhibited biofilm production was higher in MRSA strains as compared to the MSSA strains.^18^ Recently, a systematic review and meta‐analysis reported a 56.8% rate of biofilm formation among MSSA strains isolated from clinical samples in Iran.^8^ The results showed that the most common biofilm‐related genes were *fnbA*, *fnbB, icaA*, *icaD* accounted for 93.3%, 86.7%, 65.3%, and 53.3%. In contrast, the *can*, *ebp*, and *bap* genes were detected in 24%, 10.7%, and 1.3% of isolates, respectively. Similarly, analysis of 39 *S aureus* isolates recovered from urinary tract infection by Yousefi et al^23^ in Iran displayed that 69.2% were biofilm producers and the presence of *icaA*, *fnbA,* and *clfA* genes was notes in all isolates. Another similarity was in Wang et al's^44^ study from China that reported a high prevalence of *icaAD*, *fnbA*, *fnbB*, *clfAB*, and *cna* genes among their isolates. *bap* gene was only detected in one isolate. In the conformity of our finding, the low frequency of *bap* gene in *S aureus* strains was noted by some investigators.^23^, ^44^ However, our finding supports the potential of MSSA strains to persist in both hospital and community. The relationship between clonal lineage and biofilm formation has recently been reported by some investigators.^18^, ^45^ Our analysis showed that all the CC5 and the majority of CC8 strains (83.3%) showed especially strong biofilm formation ability. A similar result was in Croes et al's^18^ study from the Netherlands that reported a high prevalence of CC8, CC1, CC5, CC22, CC30, and CC45 among strong biofilm producers. Their results also displayed that CC8 was a predisposing factor for strong biofilm formation. In Malaysia, CC8 (53.3%), followed by CC1 (20%), CC22 (16.7%), and CC7 (10%) were the most common biofilm producers.^46^ Differences in biofilm formation abilities in clonal lineages of MSSA may be attributed to unique combinations of surface‐associated and regulatory genes.^18^

As illustrated in Table 1, four different types, namely CC8/ST293 (45.3%), CC/ST22 (28%), CC/ST30 (16%), and CC/ST5 (10.7%) were detected. Although CC8/ST293 has been established as one of the main international clones of MRSA, ST239 associated with MSSA isolates was reported in China.^47^ All HLMUPR strains belonged to ST239. Our findings were consistent with those from Nigeria,^48^ Kuwait,^28^ and Ireland.^42^ A study on 121 clinical MRSA and 56 MSSA isolates in 2013 by Vali et al^3^ showed that ST239 was the most identified MSSA.

We also noticed CC22 as the second dominant MSSA genotypes (28%). Besides, 76.2% of CC22 isolates were *pvl* positive. Concordant with our study, in a study conducted by Havaei et al^2^ in Iran on 83 MSSA isolates collected between January 2010 and May 2010, it was documented that ST8, ST22, ST30, and ST6 were as the most prevalent genotypes in Iran. CC/ST22 clone is widely spread both as MSSA and MRSA in China, Kuwait, Iran, the United Arab Emirates, Japan, Korea, and Australia.^6^, ^28^, ^42^ A high prevalence of *pvl* among ST22 MSSA strains has been reported by Yuan et al^6^ (80.8%). Different virulence determinants in CC/ST22 have been reported by researchers.^6^, ^36^ Contrary to earlier data that indicated HLMUPR in ST22 strains,^28^, ^36^ our analysis confirmed a 23.8% prevalence of LLMUPR in Iran. In contrast, recently published data from China showed susceptibility to most of antibiotics among MSSA clone ST22.^6^

The presence of ST30‐MSSA, known as the Southwest Pacific clone, has been noted in Australia, the UK, Germany, Lebanon, Abu Dhabi, and Kuwait.^12^, ^18^, ^28^, ^42^ Our finding that *pvl* positive ST30 was one of the most successful and persistent clones in MSSA isolates (16%), is consistent with Havaei et al's^2^ findings. As in the present study, Otokunefor et al^49^ in the UK showed CC22, CC88, CC30, and CC1 as major sequence types among *pvl* positive MSSA isolates. Notably, resistance to fusidic acid encoded by *fusB* was also detected in three isolates that belonged to ST30. This result was consistent with those in previous reports from Shore et al^36^ in Ireland, which reported resistance to fusidic acid encoded by *fusC* in fewer than half of the tested isolates.

We noted a low prevalence of ST5 (10.7%) with a high biofilm‐forming ability (62.5%) in this study. Similar observations but with different prevalence rates have been previously reported from Iran,^2^ Kuwait,^3^ Russia,^10^ Belgium,^20^ and China.^47^ Current evidence showed a high rate of resistance to PEN, GEN, TET, ERY, CIP simultaneously. These results correspond to previous report Ireland by Shore et al^36^ which indicated resistances to trimethoprim and tetracycline among CC5 isolates at a high level. Contrary to earlier studies, which indicated resistance to mupirocin in ST5 isolates, our observation does not confirm resistance to mupirocin in these isolates.^50^

In summary, this study highlights the dominant genetic lineages of MSSA isolates with reduced susceptibility to antibiotics and strong biofilm formation ability. High prevalence of MDR pattern and strong biofilm formation highlighted more attention of clinicians for rational prescription of antibiotics. Our study advocates for the continuous monitoring of the genotypes of MSSA isolates to keep track of the emerging CCs. There is a need for continued surveillance of MSSA infections in hospitalized patients.
